# Evaluation of multidrug resistance-1 gene C>T polymorphism frequency in patients with asthma

**DOI:** 10.6061/clinics/2015(10)02

**Published:** 2015-10

**Authors:** Ümran Toru, Ceylan Ayada, Osman Genç, Zehra Yaşar, Server Şahin, Emre Taşkın, İsmet Bulut, Murat Acat

**Affiliations:** I Dumlupınar University, Faculty of Medicine, Department of Chest Diseases, Kütahya, Turkey; II Dumlupınar University, Faculty of Medicine, Department of Physiology, Kütahya, Turkey; III Abant İzzet Baysal University, Faculty of Medicine, Department of Chest Diseases, Bolu, Turkey; IV Dumlupınar University, Faculty of Medicine, Department of Medical Biology, Kütahya, Turkey; V Karabük University, Faculty of Medicine, Department of Medical Biology, Karabük, Turkey; VI Süreyyapaşa Chest Diseases and Thoracic Surgery Training and Research Hospital, Department of Allergy and Immunology, İstanbul, Turkey; VII Aydın General Secretary of the Union of Public Hospitals, Aydın, Turkey

**Keywords:** Multidrug resistance-1 gene, polymorphism, p-glycoprotein, asthma, inflammation, oxidative stress

## Abstract

**OBJECTIVES:**

Asthma is a chronic inflammatory lung disease characterized by bronchial hyperresponsiveness and airflow obstruction. Genetic and oxidative stress factors, in addition to pulmonary and systemic inflammatory processes, play a pivotal role in the pathogenesis of asthma. The products of the multidrug resistance-1 gene protect lung tissue from oxidative stress. Here, we aimed to evaluate the association between the multidrug resistance-1 gene C>T polymorphism and asthma with regard to oxidative stress-related parameters of asthmatic patients.

**METHODS:**

Forty-five patients with asthma and 27 healthy age-matched controls were included in this study. Blood samples were collected in tubes with ethylenediaminetetraacetic acid. DNA was extracted from the blood samples. The multidrug resistance-1 gene polymorphism was detected by polymerase chain reaction and a subsequent enzyme digestion technique. The serum levels of total oxidant status and total antioxidant status were determined by the colorimetric measurement method.

**RESULTS:**

The heterozygous polymorphic genotype was the most frequent in both groups. A significant difference in the multidrug resistance-1 genotype frequencies between groups indicated an association of asthma with the TT genotype. A significant difference between groups was found for wild type homozygous participants and carriers of polymorphic allele participants. The frequency of the T allele was significantly higher in asthmatic patients. The increase in the oxidative stress index parameter was significant in the asthma group compared with the control group.

**CONCLUSIONS:**

The multidrug resistance-1 gene C/T polymorphism may be an underlying genetic risk factor for the development of asthma via oxidant-antioxidant imbalance, leading to increased oxidative stress.

## INTRODUCTION

Multidrug resistance (MDR) genes constitute a class of genes that play a critical role in multiple drug resistance in eukaryotic cells 1. The MDR-1 gene is located on chromosome 7q21 and plays a role in cellular regeneration 2,3. It has been reported that products of the MDR-1 gene, such as multidrug resistance-associated protein-1 (MRP1), P-glycoprotein (P-gp), and lung resistance-related protein (LRP), act as anti-oxidants and protect lung tissue against oxidative stress 4.

Asthma is a chronic inflammatory lung disease characterized by smooth muscle contraction, variable airflow obstruction, mucus hypersecretion and bronchial hyperreactivity associated with airway remodeling 5,6. Oxidative stress is one of the clearly identified pathophysiological mechanisms of this disease 5. Oxidant-antioxidant imbalances lead to pathophysiological effects associated with asthma, such as vascular permeability, mucus hypersecretion, smooth muscle contraction, and epithelial shedding. It has been previously shown that oxidant-antioxidant imbalance is associated with asthma 7, and it is also known that asthma has a strong genetic component 8.

No studies have previously investigated the relationship between MDR-1 gene polymorphisms and asthma. We aimed to investigate the association between the MDR-1 gene C>T polymorphism and asthma with regard to clinical parameters and oxidative stress indices in asthmatic patients.

## MATERIALS AND METHODS

### Participants

Forty-five patients with asthma, who presented to Dumlupınar University Medical Faculty, Department of Chest Diseases, Kütahya (a city located in the Aegean part of Turkey) and Yedikule Chest Diseases and Thoracic Surgery Training and Research Hospital, Department of Chest Diseases, İstanbul (a city located in the Marmara-northern-west region of Turkey), and 27 healthy age-matched control participants were included in this study. The asthma diagnosis was established on the basis of the criteria proposed by the Global Initiative for Asthma (GINA) guidelines. All participants were ethnically Caucasian. Individuals who had comorbidities were excluded from the study. All procedures were explained to the subjects, and written informed consent was obtained from all participants. The study protocol conformed to the ethical guidelines of the Declaration of Helsinki and was approved by the Clinical Research Ethics Committee of Abant İzzet Baysal University.

Both groups were evaluated by several clinical parameters, as follows: age; gender; body mass index (BMI; kg/m^2^); asthma control test (ACT) score; pulse; oxygen (O2) saturation; systolic and diastolic blood pressure; forced vital capacity (FVC; ml, %); forced expiratory volume in 1 second (FEV1; ml, %); FEV1/FVC; forced expiratory flow at 25-75% of the FVC (FEF25-75; %); and peak expiratory flow (PEF; ml, %). All individuals were assessed by the criteria according to the asthma control test to calculate the ACT score. For this purpose, the patients were asked the following questions: in the past 4 weeks 1) How often did your asthma limit you at work, school or at home?; 2) How often have you had shortness of breath?; 3) How often did your asthma symptoms (wheezing, coughing, shortness of breath, chest tightness or pain) wake you up at night or earlier than usual in the morning?; 4) How often have you used your rescue inhaler or nebulizer medication?; and 5) How would you rate your asthma control during the past 4 weeks? The patients scored each question from 1 to 5. The ACT scores were grouped as well controlled (score of 25), partly controlled (scores 20-24) and uncontrolled (score ≤19) 9.

### Total Antioxidant Status-Total Oxidant Status Analysis and OSI Calculation

Blood samples were collected in tubes without ethylenediaminetetraacetic acid (EDTA). After centrifugation, serum from each individual was stored at -80°C until ELISA analysis. Serum levels of the total oxidant status (TOS)- total antioxidant status (TAS) were determined by the colorimetric measurement method (Ral Assay Diagnostics). The oxidative stress index (OSI) was calculated according to the following formula 10: OSI (arbitrary unit)=TOS (µmol H_2_O_2_ Equiv./L) /TAS (mmol. Trolox Equiv./L).

#### Genotyping

##### Deoxyribonucleic acid isolation

Blood samples from 72 participants (45 asthma, 27 control) were collected in tubes with EDTA. DNA was isolated from peripheral blood leukocytes using a standard phenol/chloroform extraction method.

#### Polymerase chain reaction

Polymerase chain reaction (PCR) was used to detect the C3435T single nucleotide polymorphism (SNP). A PCR assay using the forward primer MDR1F 5'-TGC TGG TCC TGA AGT TGA TCT GTG A AC-3' and the reverse primer MDR1R 5'-ACA T TA GGC AGT GAC TCG ATG A AG GCA-3' was performed with 10× buffer, 1.5 mM MgCl2, 0.2 mM of each dNTP and 1 U Taq DNA polymerase 11. PCR amplification consisted of an initial denaturation for 2 min at 94°C followed by 35 cycles of denaturation at 94°C for 30 s, annealing at 60°C for 30 s, and extension at 72°C for 30 s. Terminal elongation was performed at 72°C for 4 min. The digestion of a 248-bp PCR product with the restriction enzyme MboI for 2 h at 37°C followed this step. The digested products were separated on a 3% agarose gel with ethidium bromide. Subsequently, the restriction fragments were identified using the UVI Gel Documentation system. The fragments obtained were 238 bp for the T/T genotype, 172 bp and 60 bp for the C/C genotype, and 238 bp, 172 bp and 60 bp for the C/T genotype 12.

#### Statistical analysis

Statistical analyses were performed using the SPSS (Statistical Package for Social Sciences, Chicago, IL, USA) 16.0 package program. Clinical and TAS-TOS-OSI parameters are presented as the mean±standard error of the mean (SEM). The significance of the observed genotype frequencies was evaluated according to the Hardy-Weinberg rule by comparison of the expected genotype frequencies. Hardy-Weinberg equilibrium was evaluated by the chi-square test. Chi-square analysis was used to test the association between asthma and the C3435T polymorphism of the MDR1 gene. A Mann-Whitney U test was used to compare the means of TAS, TOS and OSI parameters between groups. ANOVA was used to determine differences in the TAS, TOS and OSI parameters between genotypes in both groups. The odds ratio for the T allele between the groups was calculated by the chi-square test. All *p* values <0.05 were considered significant.

## RESULTS

No significant difference was observed for the clinical parameters between the groups (Table 1). In the asthma and control groups, the most frequent genotype was the heterozygous CT (n=27 and n=14, respectively). In both the asthma and control groups, homozygous wild type and homozygous polymorphic genotypes were less frequent. The most remarkable difference between the groups in terms of genotype frequency was that TT was observed in 9 patients in the asthma group and 1 in the control group. The distribution of the frequencies of the genotype for the MDR-1 gene C/T polymorphism in the asthma and control groups was compatible with the Hardy-Weinberg equilibrium (*p*>0.05; Table 3). The frequencies of MDR-1 genotypes in asthmatic patients and in control subjects are shown in Table 2. The distribution of MDR-1 genotypes were found to be 20.0% 9 for CC, 60.0% 27 for CT, and 20.0% 9 for TT in the asthma group and 44.4% 12 for CC, 51.9% 14 for CT, and 3.7% 1 for TT in the control group. The difference of the MDR-1 genotype frequencies between groups indicates an association of asthma with the TT genotype (X^2^=6,881; df=2; *p*=0,032; Table 3).

When the genotype frequency of homozygous polymorphic participants and wild type participants in the asthma group were compared with controls, there was no significance, although the level of significance was very close to the alpha level (X^2^=3.747; df=1; *p*=0.053). An association between groups was found when a similar comparison was made between wild type homozygous participants and polymorphic allele participants (odds ratio for TT=3.19; 95% CI=0.11-0.902; *p*=0.027; Table 3).

The allele frequencies for the MDR-1 gene in asthmatic patients and control subjects are shown in Table 2. The distribution for the MDR-1 gene C alleles was 50.0% 45 in the asthma group and 70.37% 38 in the control group. The distribution for the T alleles was 50.0% 45 in the asthma group and 29.63% 16 in the control group. There was a significant difference between the groups regarding allele frequency (X^2^=5.736; df=1; *p*=0.017; Table 3). The frequency of the T allele was significantly higher in asthma patients than in the control group (odds ratio for TT=3.2; 95% CI=1.1-9.2; *p*=0.027).

### Serum levels of TAS and TOS

**Figure 1:**
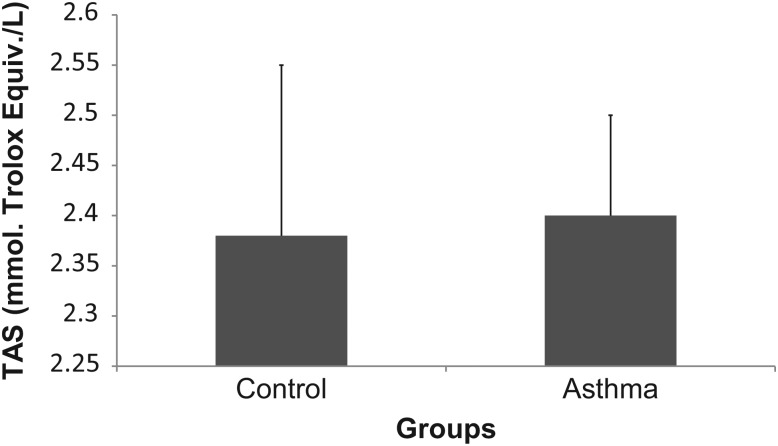
Serum levels of TAS in the control and asthma groups.

The serum levels of TAS in the asthma and control groups were as follows: 2.40±0.1 mmol. Trolox Equiv/L, 2.38±0.17 mmol. Trolox Equiv/L. No significant difference was found between the groups in terms of TAS (*p*=0.793; Figure 1).

**Figure 2:**
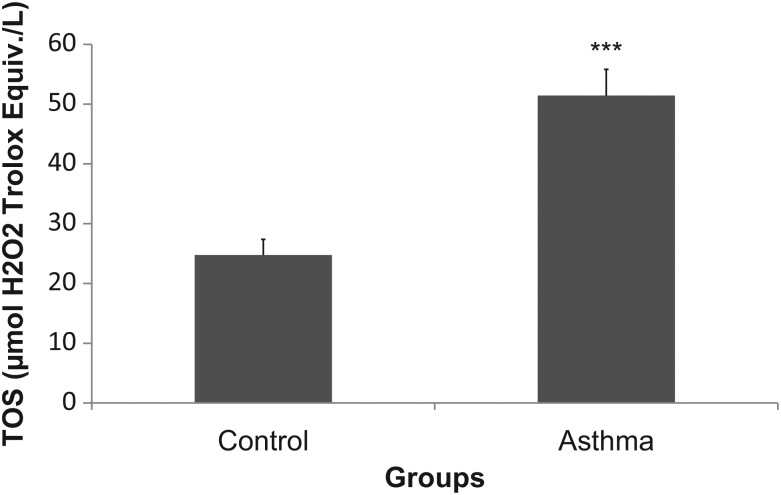
Serum levels of TOS in the control and asthma groups. ***, The significance between the control and asthma group, *p*<0.005 (Mann Whitney U test).

The serum levels of TOS in the asthma and control groups were as follows: 51.44±4.38 µmol H_2_O_2_ Trolox Equiv./L, 24.76±2.63 µmol H_2_O_2_ Trolox Equiv./L. The increase in the serum level of TOS in the asthma group was significant compared with the control group (*p*=0.000; Figure 2).

**Figure 3:**
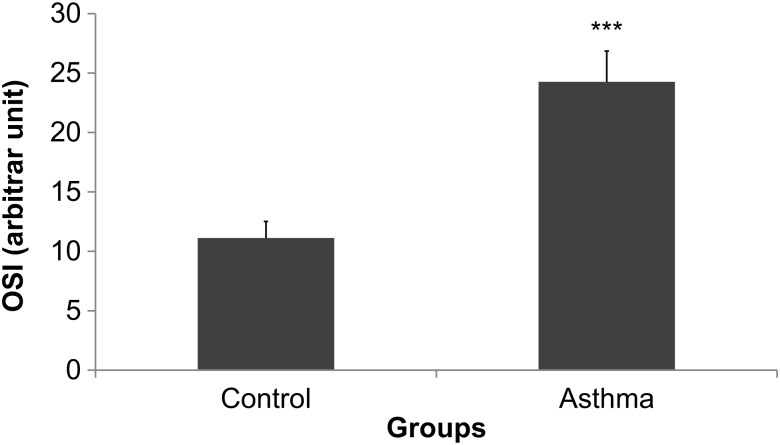
The oxidative stress index (OSI) of the L, I/R, O3+L and O3+I/R groups. ***, The significance between the control and asthma group, *p*<0.005 (Mann Whitney U test).

The OSI parameters in the asthma and control groups were as follows: 24.26±2.58 TOS/TAS, 11.12±1.39 TOS. The increase in the OSI parameter was significant in the asthma group compared with the control group (*p*=0.000; Figure 3).

In both the asthma patients and the control groups, no significant difference was observed among the three genotypes in terms of TAS, TOS and OSI (*p*>0.05).

## DISCUSSION

Asthma is a heterogeneous disorder, and a combination of environmental and genetic factors play a role in the pathogenesis of this disease 5,13. Although chronic inflammation and oxidative stress are the key mechanisms in the development of asthma, it is now known that the vast majority of asthma cases are caused by interactions between genetic and environmental risk factors 14-17. Furthermore, recent studies have revealed that multiple gene loci are involved in the etiology of asthma, and SNPs may be associated with the development of asthma 5,18.

To date, over 50 SNPs have been reported for the MDR-1 gene, and the C3435T SNP located in exon 26 of the MDR-1 gene has been shown to be associated with P-gp levels 1,19. P-gp, one of the products of the MDR-1 gene, is a transmembrane protein that acts as an ATP-driven efflux pump 1,2,20. This pump prevents the intracellular accumulation of toxic substances, drugs and metabolites by effluxing them from intracellular to extracellular areas 21,22. Therefore, P-gp has a protective role against oxidative stress by playing a role in combating the toxic effects of endogenous or exogenous irritant substances and in the removal of oxidative stress metabolites 23,24. It has been reported that the 3435C>T polymorphism of the MDR-1 gene results in a decreased expression of P-gp in T/T homozygote individuals compared with C/C homozygotes. Furthermore, a decreased expression of intestinal P-gp in T/T homozygote subjects was shown in a study by Hoffmeyer et al. 25. In another study by Dogan et al. 1, it was shown that both MDR-1 mutant homozygous (TT) and heterozygous (CT) polymorphisms were significantly more frequent in patients with chronic obstructive pulmonary disease (COPD). In a recent study, Toru et al. 26 reported an increase in the frequency of the TT genotype of the MDR-1 gene in COPD patients and suggested that the MDR-1 gene C/T polymorphism may play a role in COPD development. Today, it is well known that both COPD and asthma are characterized by chronic inflammation and remodeling of the airways 27,28. Moreover, a common pathogenetic basis for asthma and COPD is well defined based on overlapping clinical characteristics and the association of genes common for both of these diseases 29. In our study, an increased frequency of the TT genotype was observed in patients with asthma. We suggest that this increase may result in an increased exposure to oxidative stress and may be an underlying risk factor for asthma development. In addition, our study describes for the first time the frequency of the MDR-1 gene C>T polymorphism in asthma. It would be useful to show tissue P-gp expression in the study participants using PCR, in-situ hybridization, dot blot or immunohistochemical methods, but this step could not be performed because of technical issues; this is a limitation of our study.

Organisms are protected against oxidative stress via enzymatic and non-enzymatic antioxidative mechanisms, and normally, there is a balance between the rates of free radical formation and their removal by antioxidant enzymes and molecules 30,31. Therefore, the oxidative status of biological samples is accepted as an indicator of oxidative stress. In this regard, the measurement of TOS, TAS and OSI are the most common procedures that are reported to evaluate oxidative stress 30,32. In our study, we showed that the TT genotype of the MDR-1 gene with the C3435T polymorphism is associated with asthma. Additionally, we found that the blood levels of TOS and OSI were significantly higher in this genotype, which led to higher oxidative stress in asthmatic patients than in the control group. Therefore, the TT genotype may have an effect on the phenotype of the MDR-1 protein and may lead to increased oxidative stress. We can conclude that at least a proportion of this increase in oxidative stress in asthma patients may arise from the functional C3435T polymorphism of the MDR1 gene.

Finally, we can conclude that an increased T allele frequency in the MDR-1 gene may result in an increased exposure to oxidative stress in asthmatic patients. Thus, the MDR-1 gene C/T polymorphism may play a role in the development of asthma as a result of oxidant-antioxidant imbalance in favor of increased oxidative stress. However, further studies are needed to support our results and clarify the role of the MDR-1 gene polymorphism in asthma.

## Figures and Tables

**Table 1:** -Comparisons of characteristics among the genotypes in asthmatic patients.

	MDR C3435T polymorphism		
	CC	CT	TT
Age, years	39.44 ± 16.85	41.92 ± 14.18	42.44 ± 14.1
Female	19	32	9
Male	2	9	1
BMI (kg/m^2^)	29.59 ± 5.27	27.99 ± 5.91	14.44 ± 6.67
ACT (points)	15.44 ± 14.14	16.33 ± 4.53	15.78 ± 5.23
Pulse	85.12 ± 8.06	87.54 ± 12.8	87.00 ± 2.58
Saturation (%)	97.75 ± 1.03	97.31 ± 1.78	96.80 ± 1.79
SBP (mm Hg)	116.67 ± 14.14	116.67 ± 14.41	121.40 ± 14.47
DBP (mm Hg)	77.44 ± 10.19	76.89 ± 12.66	74.60 ± 8.35
FVC (%)	72.17 ± 18.70	84.25 ± 16.70	81.78 ± 13.36
FEV_1_ (%)	71.16 ± 22.28	79.98 ± 18.94	78.39 ± 16.20
FEV_1_/FVC (mL/mL)	82.90 ± 15.63	77.63 ± 12.42	79.85 ± 10.46
FEF 25-75 (%)	59.71 ± 37.23	60.30 ± 27.56	40.06 ± 29.31
PEF (%)	68.93 ± 29.51	64.54 ± 18.74	66.1 ± 27.33
TAS mmol. Trolox Equiv./L	2.43 ± 1.05	2.1 ± 1.02	3.09 ± 1.14
TOS mmol. Trolox Equiv./L	42.9 ± 20.64	51.2 ± 28.7	60.6 ± 32.6
OSI TOS µmol/l / TAC µmol of Trolox X 100	2018 ± 1412	2756 ± 1920	1988 ± 866

ACT: Asthma control test, SBP: Systolic blood pressure, DBP: Diastolic blood pressure, FVC: Forced vital capacity, FEV1: Forced expiratory volume in one second, FEF25-75: Forced expiratory flow at 25-75% of the FVC, PEF: Peak expiratory flow, TAS: Total antioxidant status, TOS: Total antioxidant status, OSI: Oxidative stress index.

**Table 2:** -Hardy-Weinberg equilibrium of the MDR gene C/T polymorphism.

		Asthma		Control	
	Allele	Expected	Observed	Expected	Observed
Common homozygotes	**CC**	11.25	9	13.37	12
Heterozygotes	**CT**	22.5	27	41.7	14
Rare homozygotes	**TT**	11.25	9	2.37	1
		*p*=0.1797		*p*=0.2059	

**Table 3:** -Genotype and allele frequencies of the *MDR* C/T polymorphism.

	Asthma		Control			
		n	%	n	%		
**Genotype Frequency**						
*MDR* C/T polymorphism						
CC	9	20.0			12	44.4
CT	27	60.0			14	51.9
TT	9	20.0			1	3.7
Total	45	100			27	100
X^2^=6.881; df=2; *p*=0.032						
CC and CT	36	80.0	26	96.3		
TT	9	20.0	1	3.7		
Total	45	100	27	100		
X^2^=3.747; df=1; *p*=0.053						
CC	9	20.0	12	44.4		
CT and TT	36	80.0	15	55.6		
Total	45	100	27	100		
X^2^=4.881; df=1; *p*=0.027						
**Allele Frequency**						
* MDR* C allele	45	50.0	38	70.37		
* MDR* T allele	45	50.0	16	29.63		
X^2^=5.736; df=1; *p*=0.017						

MDR, Multidrug resistance; df, degrees of freedom

## References

[1] Dogan OT, Katrancioglu N, Karahan O, Sanli GC, Zorlu A, Manduz S (2010). Frequency of the mdr-1 C&gt;T gene polymorphism in patients with COPD. Clinics.

[2] Gottesman MM, Hrycyna CA, Schoenlein PV, Germann UA, Pastan I (1995). Genetic analysis of the multidrug transporter. Annu Rev Genet.

[3] Israeli D, Ziaei S, Gonin P, Garcia L (2005). A proposal for the physiological significance of mdr1 and Bcrp1/Abcg2 gene expression in normal tissue regeneration and after cancer therapy. J Theor Biol.

[4] Van der Deen M, Marks H, Willemse BW, Postma DS, Müller M, Smit EF (2006). Diminished expression of multidrug resistance-associated protein 1 (MRP1) in bronchial epithelium of COPD patients. Virchows Arch.

[5] Reddy PH (2011). Mitochondrial Dysfunction and&nbsp;Oxidative Stress&nbsp;in&nbsp;Asthma: Implications for Mitochondria-Targeted Antioxidant Therapeutics. Pharmaceuticals (Basel).

[6] Bateman ED, Hurd SS, Barnes PJ, Bousquet J, Drazen JM, FitzGerald M (2008). Global strategy for asthma management and prevention: GINA executive summary. Eur Respir J.

[7] Nadeem A, Masood A, Siddiqui N (2008). Oxidant--antioxidant imbalance in asthma: scientific evidence, epidemiological data and possible therapeutic options. Ther Adv Respir Dis.

[8] Shimoda T, Obase Y, Kishikawa R, Iwanaga T Association of matrix metalloproteinase 8 genetic polymorphisms with bronchial asthma in a Japanese population. Allergy Rhinol (Providence). 2013 Fall.

[9] Global Initiative for Asthma Guideline 2014 Available from.

[10] Kosecik M, Erel O, Sevinç E, Selek S (2005). Increased oxidative stress in children exposed to passive smoking. Int J Cardiol.

[11] Turgut S, Turgut G, Atalay EO (2006). Genotype and allele frequency of human multidrug resistance (MDR1) gene C3435T polymorphism in Denizli province of Turkey. Mol Biol Rep.

[12] Akin M, Turgut S, Ayada C, Polat Y, Balci YI, Erdoğan F (2011). Relation between 3435C &gt; T multidrug resistance 1 gene polymorphism with high dose methylprednisolone treatment of childhood acute idiopathic thrombocytopenic purpura. Gene.

[13] Busse WW, Lemanske RF (2001). Asthma. N Engl J Med.

[14] Adcock IM, Ford P, Ito K, Barnes PJ (2006). Epigenetics and airways disease. Respir Res.

[15] Adcock IM, Ito K, Barnes PJ (2005). Histone deacetylation: an important mechanism in inflammatory lung diseases. COPD.

[16] Biswas SK, Rahman I (2009). Environmental toxicity, redox signaling and lung inflammation: the role of glutathione. Mol Aspects Med.

[17] Martinez FD (2007). Genes, environments, development and asthma: a reappraisal. Eur Respir J.

[18] Bouzigon E, Corda E, Aschard H, Dizier MH, Boland A, Bousquet J (2008). Effect of 17q21 variants and smoking exposure in early-onset asthma. N Engl J Med.

[19] Gümüş-Akay G, Rüstemoğlu A, Karadağ A, Sunguroğlu A (2008). Genotype and allele frequencies of MDR1 gene C1236T polymorphism in a Turkish population. Genet Mol Res.

[20] Kimchi-Sarfaty C, Oh JM, Kim IW, Sauna ZE, Calcagno AM, Ambudkar SV (2007). A &ldquo;silent&rdquo; polymorphism in the MDR1 gene changes substrate specificity. Science.

[21] Schinkel AH (1997). The physiological function of drug-transporting P-glycoproteins. Semin. Cancer Biol.

[22] Lum BL, Gosland MP (1995). MDR expression in normal tissues. Pharmacological implications for the clinical use of the P-glycoprotein inhibitors. Hematol Oncol Clin North Am.

[23] Izzotti A, Cartiglia C, Longobardi M, Balansky RM, D'Agostini F, Lubet RA (2004). Alterations of gene expression in skin and lung of mice exposed to light and cigarette smoke. FASEB J.

[24] Papp E, Gadawski I, Côté HC (2008). Longitudinal effects of thymidine analogues on mtDNA, mtRNA and multidrug resistance (MDR-1) induction in cultured cells. J Antimicrob Chemother.

[25] Hoffmeyer S, Burk O, von Richter O, Arnold HP, Brockmöller J, Johne A (2000). Functional polymorphisms of the human multidrug-resistance gene: multiple sequence variations and correlation of one allele with P-glycoprotein expression and activity in vivo. Proc Natl Acad Sci U S A.

[26] Toru U, Ayada C, Genç O, Turgut S, Turgut G, Bulut I (2014). MDR-1 gene C/T polymorphism in COPD: data from Aegean part of Turkey. Int J Clin Exp Med.

[27] Barnes PJ (2008). Immunology of asthma and chronic obstructive pulmonary disease. Nat Rev Immunol.

[28] Gelb AF, Zamel N, Krishnan A (2008). Physiologic similarities and differences between asthma and chronic obstructive pulmonary disease. Curr Opin Pulm Med.

[29] Kaneko Y, Yatagai Y, Yamada H, Iijima H, Masuko H, Sakamoto T (2013). The search for common pathways underlying asthma and COPD. Int J Chron Obstruct Pulmon Dis.

[30] Erel O (2004). A novel automated method to measure total antioxidant response against potent free radical reactions. Clin Biochem.

[31] Toprak I, Kucukatay V, Yildirim C, Kilic-Toprak E, Kilic-Erkek O (2014). Increased systemic oxidative stress in patients with keratoconus. Eye (Lond).

[32] Ferreira SM, Lerner SF, Brunzini R, Evelson PA, Llesuy SF (2004). Oxidative stress markers in aqueous humor of glaucoma patients. Am J Ophthalmol.

